# Conditions for Synaptic Specificity during the Maintenance Phase of Synaptic Plasticity

**DOI:** 10.1523/ENEURO.0064-22.2022

**Published:** 2022-05-09

**Authors:** Marco A. Huertas, Adam J. H. Newton, Robert A. McDougal, Todd Charlton Sacktor, Harel Z. Shouval

**Affiliations:** 1 Department of Neurobiology and Anatomy, University of Texas Medical School, Houston, TX 77030; 2 Yale Center for Medical Informatics, New Haven, CT 06520; 3 Department of Biostatistics, Yale School of Public Health, New Haven, CT 06520; 4 Department of Physiology and Pharmacology, SUNY Downstate Health Sciences University, Brooklyn, NY 11203; 5 Department of Physiology, Pharmacology, Anesthesiology, and Neurology, SUNY Downstate Health Sciences University, Brooklyn, NY 11203; 6 Department Electrical and Computer Engineering, Rice University, Houston, TX 77005

**Keywords:** LTP, maintenance, protein synthesis, synapse specificity, synaptic plasticity

## Abstract

Activity-dependent modifications of synaptic efficacies are a cellular substrate of learning and memory. Experimental evidence shows that these modifications are synapse specific and that the long-lasting effects are associated with the sustained increase in concentration of specific proteins like PKM*ζ*. However, such proteins are likely to diffuse away from their initial synaptic location and spread out to neighboring synapses, potentially compromising synapse specificity. In this article, we address the issue of synapse specificity during memory maintenance. Assuming that the long-term maintenance of synaptic plasticity is accomplished by a molecular switch, we carry out analytical calculations and perform simulations using the reaction-diffusion package in NEURON to determine the limits of synapse specificity during maintenance. Moreover, we explore the effects of the diffusion and degradation rates of proteins and of the geometrical characteristics of dendritic spines on synapse specificity. We conclude that the necessary conditions for synaptic specificity during maintenance require that molecular switches reside in dendritic spines. The requirement for synaptic specificity when the molecular switch resides in spines still imposes strong limits on the diffusion and turnover of rates of maintenance molecules, as well as on the morphologic properties of synaptic spines. These constraints are quite general and apply to most existing models suggested for maintenance. The parameter values can be experimentally evaluated, and if they do not fit the appropriate predicted range, the validity of this class of maintenance models would be challenged.

## Significance Statement

Synapse-specific long-lasting synaptic plasticity is essential for the ability to learn and remember. Here, we develop a biophysical model to explore the constraints on synapse specificity during the maintenance phase of synaptic plasticity. We assume that maintenance is accomplished by a local molecular switch and show that because of diffusion of molecules within dendrites, synaptic specificity might be lost. Our results demonstrate that to preserve synapse specificity, the location of the molecular switch must reside within synaptic spines. Previous work has indicated that the molecular switch is implemented at the level of translation. Therefore, our model makes the specific prediction that in potentiated synapses synaptic spines must contain polyribosomes. Our results are general and apply to the various mechanist implementations of a molecular switch, placing severe constraints on most proposed maintenance models.

## Introduction

Overwhelming experimental evidence indicates that activity-dependent modification of synaptic efficacies is the cellular substrate of learning and memory (Morris et al., 1990; Martin and Morris, 2002; Whitlock et al., 2006; Nabavi et al., 2014). Much is known about the molecular substrate of one form of synaptic plasticity, long-term potentiation (LTP). Theoretically, such synaptic plasticity must be synapse specific, the property of synapses whereby only activated synapses undergo modifications in their synaptic efficacies while neighboring synapses remain unaltered, and this is supported by extensive experimental evidence (Andersen et al., 1977; Lynch et al., 1977; Harvey et al., 2008). The experimental evidence, however, is mostly limited to the induction phase of synaptic plasticity (but see Govindarajan et al., 2011). Changes occurring at the synapse level, because of neuronal activity, include molecular alterations to the synapse machinery (Bosch et al., 2014), structural changes to dendritic spines (Matsuzaki et al., 2004; Tønnesen et al., 2014), and an increase in the synthesis of an assortment of proteins (Costa-Mattioli et al., 2009; Bosch et al., 2014). Some of these changes last for a few hours while others must endure for days or a lifetime. A long-standing problem, first raised by Crick in 1984 (Crick, 1984), is how can memories and their cellular substrate last for much longer periods of time than the lifetimes of the molecular substrates. A possible solution to this quandary is that local synaptic molecular switches can maintain stable synaptic efficacies even if their molecular substrates are transient (Lisman, 1985; Lisman and Zhabotinsky, 2001; Klann and Sweatt, 2008; Aslam et al., 2009; Jalil et al., 2015).

Over the last couple of decades an accumulation of evidence has shown that a sustained increase in the concentration of specific isoforms of PKC is associated with the long-lasting form of LTP (L-LTP; Tsokas et al., 2016; Wang et al., 2016). Chief among these PKM*ζ*, an atypical isoform of PKC, has been shown to be necessary and sufficient for some forms of long-term plasticity and memory (Sacktor et al., 1993; Drier et al., 2002; Ling et al., 2002; Wang et al., 2016; Tsokas et al., 2016). However, in mutants lacking the gene for PKM*ζ* another isoform of atypical PKC (PKC*ι* /*λ*), is upregulated and becomes necessary for maintenance. Experimental evidence indicates that polyribosomes, responsible for protein synthesis, exist both in dendrites and spines, and that the induction of LTP increases the number of them in synaptic spines (Ostroff et al., 2002, 2018; Sutton and Schuman, 2006; Bourne et al., 2007). Therefore, the likely substrate for the molecular switch that maintains memory and, at the same time, is synapse specific is implemented at the level of the synthesis of new proteins (Aslam et al., 2009), likely the synthesis of PKM*ζ* (Jalil et al., 2015). This is in line with experimental evidence that shows that inhibiting the synthesis of new PKM*ζ* with antisense prevents the formation of L-LTP and long-term memory (Tsokas et al., 2016).

Most mathematical models of maintenance are based on the concept of bistability or multistability (Lisman, 1985; Lisman and Zhabotinsky, 2001; Aslam et al., 2009; Jalil et al., 2015). This means that a synapse has a discrete set of stable states which can last indefinitely despite protein turnover and diffusion. The low stable state (down-state) corresponds to an unpotentiated synapse and the upper stable states (up-state) correspond to the synaptic efficacy after potentiation. These states are generated by the protein dynamics, and usually arise because of positive feedback. Thus far, studies of bistable switches as the basis for the maintenance phase of synaptic plasticity have concentrated on a single compartment, and have shown that switches based on positive molecular feedback can be bistable or multistable. However, products can diffuse from one switch to another and turn on an inactive switch, potentially eliminating synapse specificity during maintenance. Moreover, considering that plasticity-related proteins such as PKM*ζ* and CaMKII might be degraded slowly (van de Nes, 2012; Wang et al., 2016) and that PKM*ζ* is constitutively active (for review, see van de Nes, 2012), might produce long protein length-constants, as explained below. Such long length-constants mean that molecular switches at distant locations can potentially interact, placing a severe constraint on synapse specificity.

In this article, we address the issue of synapse specificity during maintenance, employing a computational approach based on reaction-diffusion equations in dendritic shafts and spines. We study this equation using both analytical solutions and numerically using the reaction-diffusion package of the NEURON simulation platform (Carnevale and Hines, 2006; McDougal et al., 2013, 2022). This analysis allowed us to estimate the limits of synapse specificity when the molecular switches are located in dendritic shafts or in dendritic spines and how these limits depend on the system parameters. We further examine the impact of synaptic clustering on synapse specificity, and how such partial specificity depends on cluster parameters. Our theoretical results allow us to make strong predictions that can be tested experimentally, to further support or challenge this class of maintenance models.

## Materials and Methods

We developed a spatial model consisting of a dendritic branch with a variable number of dendritic spines, and numerically calculated the steady-state spatial distribution of proteins being synthesized at various locations in the model’s volume. Proteins are synthesized in specified locations on the dendrite or in the head of dendritic spines. Furthermore, proteins can be degraded anywhere and are allowed to diffuse throughout the defined volume. Here, we describe the various elements and setup of the model. For synapse specificity of maintenance to apply, the system must meet two conditions. First, the maximum protein synthesis rate, defined as *I_o_* must be large enough such that synaptic efficacy can be bistable, and able to remain in the conditioned, up-state even when all other neighboring synapses are in the unpotentiated down-state. This condition sets the minimal value of *I_o_* which is defined as $I_{o}^{*}$. Second, it must be possible to maintain a single synapse in the down-state when all its neighbors are in the up-state. The second condition sets the minimal allowed distance between synapses.

### Molecular switch model

The model we describe is a very simple generic model. It could represent switches generated by protein synthesis, and protein degradation and diffusion. However, a similar model could also represent posttranslational modifications. Hereafter, synthesis could refer to either actual translation of new proteins, of to the posttranslational modification of proteins. Similarly, the location of a polyribosome could also refer to the location of the posttranslational process. In this switch model we assume a concentration-dependent synthesis component and simple linear degradation component. The synthesis rate depends on the protein concentration at the location of the polyribosome (Kelly et al., 2007; Costa-Mattioli et al., 2009), while the degradation rate depends on the local protein concentration throughout the volume. In general, this nonlinear positive feedback can generate bistability or multistability solutions (Lisman, 1985; Lisman and Zhabotinsky, 2001; Aslam et al., 2009; Jalil et al., 2015). In the absence of diffusion, the change in protein concentration (*c*), at one point, is described by the following equation:

(1) $$\frac{dc}{dt}=I_{o}\Theta (c-c_{\theta })-K\cdot c,$$

where *I_o_* is the maximum protein synthesis rate, *c_θ_* is an activation threshold, and *K* is the protein degradation rate. The activation function Θ is assumed to have a general sigmoid shape. In the analytical calculations presented here we will assume that Θ has a step-function form.

**Figure 1.**
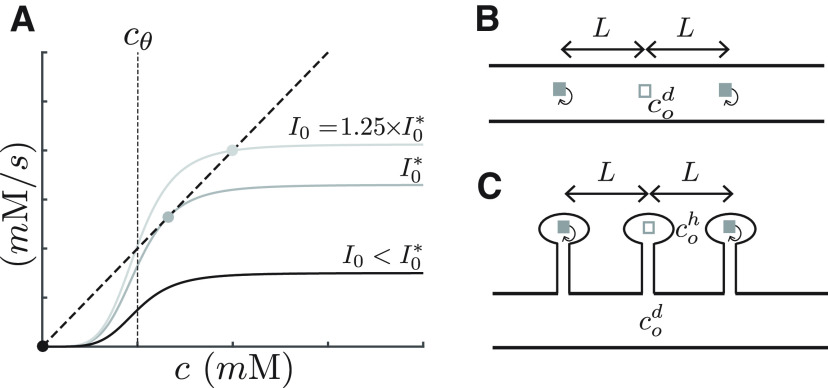
Bistable switch and model setup. ***A***, This figure graphically illustrates the source and sink terms on the right hand-side of Equation 1, and their fixed points for various values of *I_o_*. The sigmoidal shapes in gray illustrate the source term of Equation 1, which is $I_{0}\theta (c-c_{\theta })$, for various levels of *I*_0_. The diagonal dark line is the sink term (*K* ⋅ *c*). Fixed points are the intersections of the source and sink terms (points where *dc*/*dt *=* *0, so that sink = source). Stable fixed points are indicated by circles. Below $I_{o}^{*}$ there is one fixed point at *c *=* *0 (down-state), whereas for values above $I_{o}^{*}$, there is a second stable fixed point at higher concentrations (up-state). The value of $I_{o}^{*}$ is the smallest value of *I_o_* required to produce an up-state. The activation curve Θ illustrated here has a sigmoid dependency on protein concentration. ***B***, Schematic of the case of switches (polyribosomes) in the dendrite. Two active switches (gray boxes with feedback arrows) are located a distance *L* on each side of a third inactive switch (empty white box). The protein concentration at the location of the inactive switch is labeled $c_{o}^{d}$. ***C***, Similar to ***B*** for the case of switches in dendritic spine heads. The protein concentration at the location of the inactive switch is labeled $c_{o}^{h}$, whereas the concentration on the dendrite at the location of the spine is $c_{o}^{h}$.

The value of *I_o_* is chosen to guarantee a bistable steady-state solution. Figure 1*A* shows the fixed points of Equation 1 (i.e., when *dc*/*dt *=* *0) for different values of *I_o_*, while keeping the degradation rate *K* constant. As illustrated here, for values of *I_o_* below $I_{o}^{*}$ there is only one fixed point at *c *=* *0. This solution will be referred to as down- or inactive-state. For values above $I_{o}^{*}$ there is another stable solution at higher concentrations. This corresponds to the up- or active-state. For a Θ that has a step function form, the value of $I_{o}^{*}$ is simply $I_{o}^{*}=K\cdot c_{\theta }$.

We consider that for active synapses the switch is operating in the up-state of the bistable regime. In the case when Θ is represented by a step-function, the up-state lies in the saturation region, otherwise if it has a sigmoidal shape the up-state will be just below saturation. We use a maximal current magnitude of $I_{o}=fI_{o}^{*}$, with *f *>* *1, which is above the critical value. In simulations we chose *f *=* *1.25. Clearly the value of $I_{o}^{*}$ will be different for the various conditions investigated here.

### Solutions for a single isolated switch

In the case of a polyribosome in the dendrite, we replace the single compartment equation with a 1D reaction-diffusion equation of the form:

(2) $$\frac{\partial c^{d}(x,t)}{\partial t}=D\frac{\partial^{2}c^{d}(x,t)}{\partial x^{2}}+I\delta (x)-Kc^{d}(x,t),$$

where the label *d* indicates the concentration is in the dendrite, the term *δ(x)* indicates that the source term is placed in the location *x *=* *0, and $I=I_{o}\theta (c_{o}^{d}-c_{\theta })$ is the synthesis rate with a maximum value *I_o_* and $c_{o}^{d}=c^{d}(x=0)$. The steady-state solution is given by

(3) $$c^{d}(x)=\frac{\lambda I_{o}\Theta (c_{o}^{d}-c_{\theta })}{2D}\exp\left(-\frac{\left|x\right|}{\lambda }\right),$$

where $\lambda =\sqrt{D/K}$ is the characteristic length constant of the protein. Evaluating this solution at *x *=* *0, provides an expression from which to determine the value of $I_{o}^{*}$:

(4) $$I_{o}^{*}\Theta (c_{o}^{d}-c_{\theta })=\frac{2D}{\lambda }c_{o}^{d}.$$

This equation is similar to the fixed point of Equation 1 with *K* replaced by 2*D*/*λ*, and thus, as illustrated in Figure 1*A*, the value of $I_{o}^{*}$ is determined so that $c_{o}^{d}$ is in the up-state. For the step function the minimal value of *I_o_* therefore has the form: $I_{o}^{*}=(2D/\lambda )c_{\theta }$.

In the case of polyribosomes in dendritic spines, we first solved Equation 2 in each compartment representing the dendritic spine (Fig. 3). Solutions for each compartment are matched at the boundaries. Moreover, the fluxes between compartments are related through the expression $r_{a}^{2}J_{a}=r_{b}^{2}J_{b}$, where *r* is the radius and *J* is the flux in or out of the compartment. Here, we have used a simplified expression that agrees with the simulations. A more elaborated formulation can be found in Berezhkovskii et al. (2009); however, for our conditions, the expressions are identical.

### The resulting equations are combined to derive the expression


(5) $$\begin{array}{l} \frac{2D}{\lambda }c_{o}^{h}=\left\{\alpha \frac{{\left(r_{\text{neck}}/r_{\text{dend}}\right)}^{2}P}{1+(\lambda /2D){\left(r_{\text{neck}}/r_{\text{dend}}\right)}^{2}Q}-\frac{2D}{\lambda }\beta \right\}I_{o}\Theta (c_{o}^{h}-c_{\theta })\\ =AI_{o}\Theta (c_{o}^{h}-c_{\theta }) \end{array},$$

where *r*_neck_ is the radius of the spine neck, *r*_dend_ is the radius of the dendritic compartment, and *α*, *β*, *P*, and *Q* are terms containing geometrical, diffusion, and degradation parameters (see below, Parameters and auxiliary functions). This equation is used to find $I_{o}^{*}$ under the same conditions as those for Equation 4, i.e., to guarantee that the solution corresponds to the up-state.

### Model setup: multiple switches

To determine the limits of the ability of synapses to maintain their specificity during the maintenance phase, we consider the following setup. We place 2*N *+* *1 bistable switches (e.g., polyribosomes with positive feedback) equidistant from each other along the length of a dendritic branch. One switch is located at *x *=* *0 and is considered to be in the down-state while the remaining ones, separated a distance *L*, are in the up-state presumably as the result of synapses being potentiated. Figure 1*B* shows this situation for the case *N *=* *1.

Proteins synthesized by the active switch diffuse along the dendrite and can cause an increase in the concentration at the location of the inactive switch. The concentration at this location will depend on the distance *L* between them and the number of other active switches. We determine the smallest distance (*L_crit_*) at which the inactive switches remain in the down-state when N→∞. The N→∞ is the most stringent limit, and additionally, the results presented are then simpler as they are independent of the number *N*. However, the results do not strongly depend on this limit, and using a moderate value of *N* (e.g., *N *≥* *5) will cause only a small difference in the results. The value of *L_crit_*, which we refer as the critical distance, provides a measure of the distance between activated synapses that will keep an inactive synapse isolated.

A similar setup is made for the case of switches located in the heads of dendritic spines, as illustrated in Figure 1*C*. Here, the distance *L* refers to the interspine distance and thus *L_crit_* measures how close dendritic spines (and their corresponding synapses) could be from each other and still remain isolated.

### Analytical expressions for *L_crit_*

The value of *L_crit_* can be calculated analytically. In the case of switches along a dendrite, we assume that all active switches are operating in the saturation regime with a maximum synthesis rate *I_o_*. In this case the concentration at the location of the inactive switch takes the following form:

$$c_{o}^{d}=\frac{\lambda I_{o}}{2D}\;\Theta (c_{o}^{d}-c_{\theta })+2\times \frac{\lambda I_{o}}{2D}\sum_{n=1}^{n=N}e^{-\frac{nL}{\lambda }},$$

where *λI_o_*/2*D* is the maximum concentration at the location of an isolated switch (see Eq. 3). The first term in this expression represents the contribution from the inactive switch, whereas the second term is the contributions of the 2*N* active switch. The exponential function arises from the solution to the 1D diffusion equation with a constant concentration-dependent degradation rate (cf. Eq. 3). The geometric series has the closed form, resulting in the equation:

$$\frac{2D}{\lambda }c_{o}^{d}=I_{o}\Theta (c_{o}^{d}-c_{\theta })+2I_{o}\frac{e^{-L/\lambda }-e^{-(N+1)\cdot L/\lambda }}{1-e^{-L/\lambda }}.$$

In the limit of N→∞ this expression becomes:

(6) $$\frac{2D}{\lambda }c_{o}^{d}=I_{o}\Theta (c_{o}^{d}-c_{\theta })+2I_{o}\frac{e^{-L/\lambda }}{1-e^{-L/\lambda }}.$$

**Figure 2.**
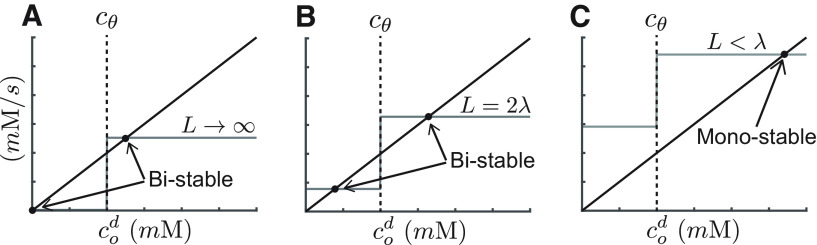
Transition from bistability to monostability as a function of interswitch distance for the case of an infinite number of sources in the dendrite. When flanking switches are active, proteins from those active switches diffuse to the location of an inactive switch, generating a basal activity even for an inactive switch. The closer the flanking switches are (small L), the larger the basal activity. Such basal activity can affect the number of possible fixed points. Panels ***A–C*** illustrate the effect of the distance between polyribosomes on the number of stable fixed points in Equation 6. The diagonal black line in the sink term as in Figure 1. The step function in each panel is the effective source term, assuming a step theta function. The additional sources effectively elevate the source term because of diffusion of protein from the additional sources. The closer those sources are, the larger the elevation of the effective source term. Fixed points are indicated by black circles and correspond to the intersection between the activation curve (gray step-function) and decay rate curve (solid black line). The three examples correspond to different distances between switches: (***A***) L→∞, (***B***) *L *=* *2*λ*, and (***C***) *L* < *λ*. The system undergoes a transition from bistability to monostability between (*L *=* *2*λ*) and (*L* ≤ *λ*).

Note, that the difference between the two equations above is the term $e^{-N\cdot L/\lambda }$. When switches reside in dendrites, this term is very small even for moderate values of *N*. For example, a solution for *N *=* *5 differs from the infinite limit by a few percent. We solve this equation for $c_{o}^{d}$, the concentration at the location of the inactive switch. The solutions of Equation 6 for various values of *L* and a step function Θ are graphically illustrated in Figure 2. As *L* becomes smaller the solutions undergo a transition from bistability to monostablilty. The value at which this transition occurs defines *L_crit_* because the only solution possible at smaller distances will render all switches in the up-state.

### Analytical expressions for *L_crit_* with switch in spines

A similar analysis can be done when switches are located in the head of dendritic spines. The logic is similar but the derivations more complex. In this case we are interested in the concentration in the spine head ( $c_{o}^{h}$), at the location of the protein synthesis source term. Because of diffusion through the dendritic spines the concentration *c^h^* depends on both *I_o_* and $c_{o}^{d}$, the concentration in the dendrite at the location where the spine neck connects with the dendrite (Fig. 1*C*)

(7) $$c_{o}^{h}=\alpha c_{o}^{d}+\beta I_{o}\Theta (c_{o}^{h}-c_{\theta }),$$

where *α* and *β* are parameters determined by the spine geometry, the diffusion constant *D* and the characteristic length constant *λ* (see below, Parameters and auxiliary functions). The label *o* serves to identify these quantities as pertaining to the spine at *x *=* *0. The value of $c_{o}^{d}$ is determined by superimposing the contributions from the inactive spine and the contributions from the remaining 2*N* spines. The final expression becomes, in the limit of N→∞,

(8) $$\frac{2D}{\lambda }c_{0}^{h}=AI_{o}\Theta (c_{0}^{h}-c_{\theta })+BI_{o}\frac{e^{-L/\lambda }}{1-e^{-L/\lambda }},$$

where A and B are constant functions of the geometry of the spines, the diffusion constant *D* and the length constant *λ* (see below, Parameters and auxiliary functions). This equation resembles Equation 6 above, and thus we can determine the critical value *L_crit_* similarly, as depicted in Figure 2.

Equations 6, 8 are the main equations used for this study. Note that on the left hand-side of these equations there are two terms. The first term arises from the current of the central synapse, and the second term from all the flanking potentiated synapses. Formally, for any shape of the activation function *θ*, the value of *L_crit_* can be obtained by the following procedure. First, we need to identify the critical current to sustain an isolated synapse. This is done by ignoring the second term on the left hand-side of the equation, and carrying out a single parameter bifurcation analysis in terms of *I*_0_. The transition point between a single solution (monostable) and three solutions (bistable) is the critical value of *I*_0_, termed $I_{0}^{*}$. After this we set $I_{0}=f\cdot I_{0}^{*}$. Given this we then consider to complete equation and carry out bifurcation analysis in terms of *L*, to obtain *L_crit_*. The limiting case of the activation function being a step function is much simpler and allows for analytical solutions for *L_crit_*.

### Critical distance with a finite number of sources

In addition to our model of many infinite sources surrounding a single unpotentiated source, we also consider a limited number of active sources. In this case we have a long dendritic branch with *N* sources of which *n* are potentiated. As before, given the geometry of the dendrite and length scale *λ* for the protein, there exists a critical distance *L_crit_*. If the sources are closer together than this critical distance, then all sources will become potentiated, if the sources are further apart than *L_crit_*, the *N* – *n* sources will not become potentiated. We place the unpotentiated sources from origin extending to the left at multiples of distance *L*, i.e., −(N−n−1)L,...,−L,0 with the potentiated sources extending from *L* to the right, *L*,2*L*,…,*nL*. The steady-sate concentration at the origin is similar to this equation:

$$\frac{2D}{\lambda }c_{o}^{d}=I_{o}\sum_{k=0}^{N-n-1}\Theta (c^{d}(kL)-c_{\theta })e^{-kL/\lambda }+I_{o}e^{-L/\lambda }\left(\frac{1-e^{-nL/\lambda }}{1-e^{-L/\lambda }}\right).$$

To determine the critical distance, it is sufficient to consider only the unpotentiated source at the origin; if this source potentiates, so will the others.

A similar modification to Equation 8 gives the steady-state when the sources are in spine heads:

(9) $$\frac{2D}{\lambda }c_{0}^{h}=AI_{o}\Theta (c_{0}^{h}-c_{\theta })+\frac{1}{2}BI_{o}\left(e^{-L/\lambda }\left(\frac{1-e^{-nL/\lambda }}{1-e^{-L/\lambda }}\right)+\sum_{k=1}^{N-n-1}\Theta (c_{k}^{h}-c_{\theta })e^{-kL/\lambda }\right),$$

where A and B are determined by the geometry of the spine and dendrite and $c_{k}^{h}$ is the concentration in the *k*th spine head. As before, *L_crit_* is determined by finding the maximum distance between spines such that the unpotentiated spine at the origin becomes potentiated.

### Analytical expression if diffusion in potentiated synapses is different from unpotentiated synapses

Here, we allow for the possibility that diffusion rates and length constants in spine compartments differ from those in dendrites. We also allow for the possibility that diffusion in potentiated synapses is different from diffusion in unpotentiated synapses. We will assume for simplicity that for each spine, the diffusion in the neck and the head as well as the turnover are the same. In the dendrites, as before, the diffusion coefficient and the length constant are *D* and *λ*, in spines they are *D_x_* and *λ_x_* where *x *=* a* or *i*, depending on whether this is an active (potentiated) of inactive (unpontentiated) spine, respectively. Using analogous methods to those used above, the critical value of the current for an isolated spine with a step function switch is:

(10) $$I_{0}^{*}=\frac{2Dc_{\theta }}{\lambda A_{x}},$$

where $A_{x}$ is defined in section. Here, we can assume that the critical value is calculated either for the active or inactive parameters, and this will determine whether *x *=* a* or *x *=* i*. The choice of these options and their consequences are described in Results. The actual current used is assumed to be $I_{0}=f\cdot I_{0}^{*}>I_{0}^{*}$. From the bistability condition of a single spine flanked by an infinite number of spines on both sides, we obtain the equation for the concentration in an inactive head defined as $c_{h}^{i}$:

(11) $$c_{h}^{i}=(\frac{\lambda }{2D})B_{a}I_{0}\frac{e^{-L\lambda }}{1+e^{\frac{-L}{\lambda }}}.$$

By combining these two equations we get the equation for *L_crit_* in this case which is:

(12) $$L_{crit}=\lambda \ln\left(1+f\frac{B_{a}}{A_{x}}\right).$$

### Computational model and simulations

**Figure 3.**
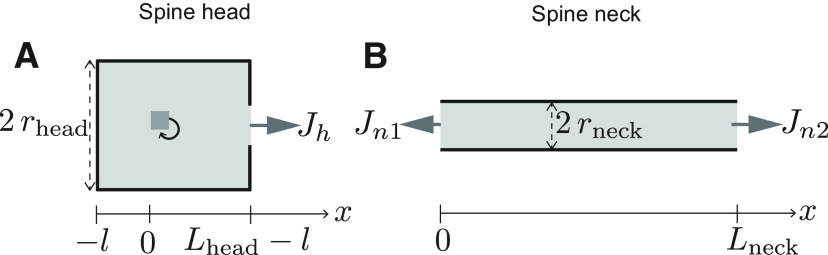
Geometry of spine. ***A***, ***B***, Schematics of the compartments representing the spine head and neck, respectively. Compartments are assumed to be cylindrical in shape with diameters 2*r*_head_ and 2*r*_neck_, and lengths *L*_head_ and *L*_neck_, respectively. The switch (gray box with a feedback arrow) is located a distance *l* from the sealed end of the spine head compartment. The quantities *J_h_*, *J_n_*_1_, and *J_n_*_2_ represent protein fluxes per unit area out and in of the respective compartments.

Simulations were performed using the reaction-diffusion package (McDougal et al., 2013; Newton et al., 2018) in NEURON (Carnevale and Hines, 2006) to solve for the steady-state concentration distribution of protein. Proteins were allowed to diffuse and degrade in a volume representing a dendritic branch with a variable number of dendritic spine compartments. The dendritic branch was considered as a cylinder of diameter *d*_dend_ and length *L*_dend,_ and the spine geometry is illustrated in Figure 3. All the code is written in Python. The parameters used in the simulations performed are given in Table 1. The characteristic length constant, *λ* (see Eq. 4), which depends both on the diffusion and the degradation rate, is explored as a variable parameter in this model. The length of the dendritic branch was adjusted depending on the value of *λ* to ensure the boundary conditions did not substantially affect the model. The number of segments were chosen to give a spatial grid of 1 μm. In the simulations the activation curve Θ was modeled as a steep Hill function,

(13) $$\Theta (c,c_{\theta })=\frac{c^{n}}{c^{n}+c_{\theta }^{n}}.$$

**Table 1:** Model parameters used in simulations

Model parameters			
Diffusion coefficient	*D*	1 × 10^–3^	μm^2^/ms
Characteristic length constant	*λ* †	Variable	μm
Hill function exponent	*n*	40*	—
Activation threshold	*c_θ_*	2	mm
Diameter dendritic branch	2*r*_dend_	5	μm
Length dendritic branch	*L*_dend_	Variable	μm
Number of segments dendritic branch	*N*_dend_	Variable	—
Diameter spine head	2*r*_head_	1	μm
Length spine head	*L*_head_	1	μm
No. segments spine head	*N*_head_	5	—
Diameter spine neck	2*r*_neck_	0.2	μm
Length spine neck	*L*_neck_	2	μm
No. segments spine neck	*N*_neck_	25	—

^†^the expression for *λ* is given below Equation 3 in terms of the degradation constant *K*.

*in Figure 5C, *n *=* *300 is used.

The values of some of the parameters are altered in several figures, as indicated in those figures.

**Figure 4.**
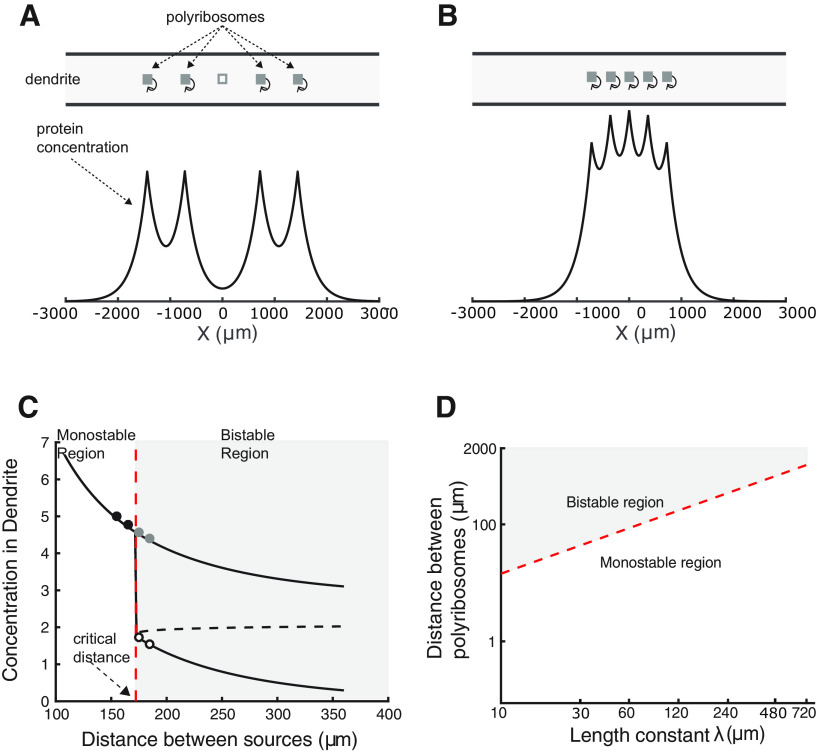
Transition between “up” and “down” states for switches located in dendrites. ***A***, Upper panel illustrates an arrangement of active switches (gray-filled boxes) on both sides of an inactive one (open box) along a dendrite. Lower panel shows a steady-state protein concentration profile. The concentration peaks at the location of the active switches. ***B***, Similar to ***A*** but with switches located closer to each other. Here, the central switch has become active. ***C***, Example showing the transition from a bistable to a monostable mode for the central switch as the distance between switches decreases. Black solid and dashed lines represent stable and unstable fixed points of Equation 6, respectively, as a function of switch separation. Circles correspond to simulations performed near the transition boundary (vertical red dashed line). Filled symbols correspond to up-state and open circles to down-state solutions. The bistable state means that synapse specificity can be maintained, whereas a monostable state means the central synapse can only reside in a single state, which is dictated by its neighbors. ***D***, Diagram showing the dependency of critical distance on the characteristic length constant *λ*. Only the bistable region has synapse specificity.

**Figure 5.**
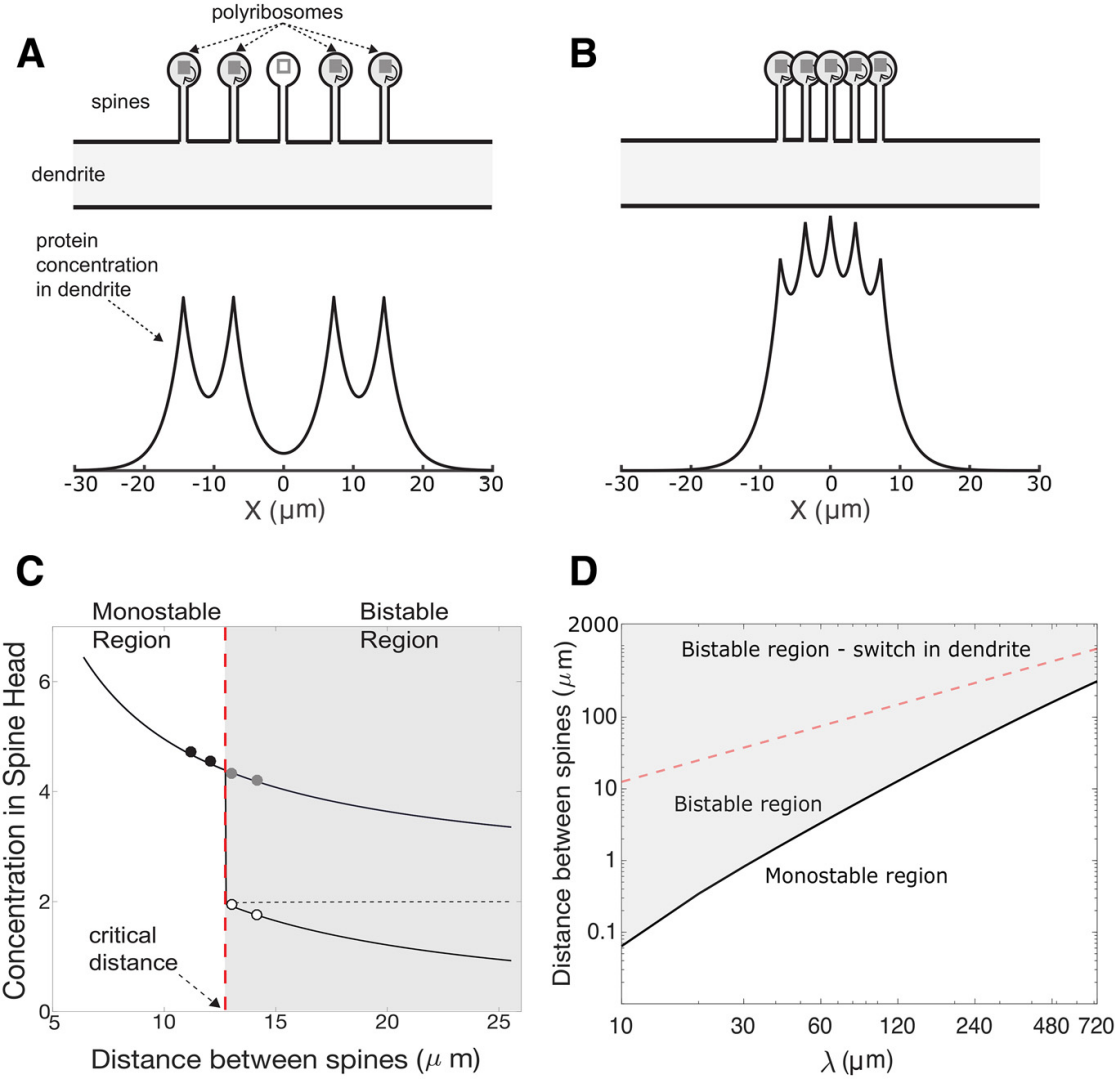
Transition between up- and down-states for switches located in the head of dendritic spines. ***A***, Upper panel illustrates an arrangement of active switches (gray-filled boxes) on both sides of an inactive one (open box), all located in the heads of dendritic spines distributed along a dendrite. Lower panel shows the steady-state protein concentration profile as measured in the dendrite. The concentration peaks at the location of the spines with active switches. ***B***, Similar to ***A*** but with spines placed closer to each other. In this situation the switch in the central spine has become active. Compare with Figure 4. ***C***, Example showing the transition from a bistable to a monostable mode for the switch in the central spine (compare with ***A***) as the distance between spines decreases. Black solid and dashed lines represent stable and unstable fix points of Equation 8 as a function of spine separation. Circles correspond to 1D simulations performed near the transition (vertical red dashed line). Filled symbols correspond to up-state and open circles to down-state solutions. Both numerical and simulation results are with a hill function exponent of *n *=* *300. ***D***, Diagram showing the dependence of the critical distance on the characteristic length constant *λ*. Red dashed curve represents the critical distance for switches in spine heads. Light red dashed curve is the result when switches are in the dendrite (compare Fig. 4*D*). These results are for *λ* = 20 μm and spine neck length of 2 μm. Note that the bistable region when synaptic switches are in spines is much larger, implying a larger parameter range for synapse specificity.

Different coefficients (*n*) are used throughout, when trying to compare to results of the step function we use a very steep exponent of *n *=* *300, in most other cases we use *n *=* *40. Simulations that approximate the limiting case of an infinite number of switches in dendrites or spines (Figs. 4*C*, 5*C*, 10) were performed using a finite number of sources. The number was chosen so that no significant change was observed in the concentration at the location of the inactive synapse when an additional pair of sources was added. This number was different for different values of *λ*. Typical values of *λ* were between 10 μm and 300 μm.

The spines were all connected along the top of the dendrite, and this could lead to a gradient of protein within the dendrite that would not be captured by the 1D simulations described above. To validate our 1D approximation we constructed a hybrid 1D/3D simulation, with *N *=* *5, neck length 5 μm source 284 μM/s, Hill-function exponent 40 and *λ* = 60 μm. The central three spines (the inactive spine and its two neighbors) were simulated in 3D, together with the central section of dendritic shaft from –(3/2)L to (3/2)L, voxel size of 0.125 μm^3^. The additional spines together with their dendritic shaft were modeled in 1D to provide appropriate boundary conditions.

### Parameters and auxiliary functions

Model parameters are presented in Table 1, and the terms involving geometrical, diffusional, and degradation parameters in Equations 7, 8, 5 are given here. Dendritic diffusion coefficients are defined as *D* and *λ*. The subscript *x* ∈ {*a*,*i*} denotes whether the spine is active or inactive. In cases where all diffusion coefficients and length constants are equal, the subscripts can be dropped. If a subscript does not appear, *D* or *λ* dendritic values should be used.

$$\begin{array}{l} P_{x}\;=\;\frac{\cosh(l/\lambda_{x})/\sinh(L_{\text{head}}/\lambda )}{\cosh(L_{\text{neck}}/\lambda_{x})a_{x}-\sinh{\left(L_{\text{neck}}/\lambda_{x}\right)}^{-1}}\\ Q_{x}\;=\;\frac{D_{x}}{\lambda_{x}}\frac{\sinh(L_{\text{neck}}/\lambda_{x})a_{x}}{\cosh(L_{\text{neck}}/\lambda_{x})a_{x}-\sinh{\left(L_{\text{neck}}/\lambda_{x}\right)}^{-1}}\\ a_{x}\;=\;\coth(L_{\text{neck}}/\lambda_{x})+{\left(r_{\text{neck}}/r_{\text{head}}\right)}^{2}\coth(L_{\text{head}}/\lambda_{x})\\ \alpha_{x}\;=\;\frac{\cosh(l/\lambda_{x})}{\sinh(L_{head}/\lambda_{x})\cosh(L_{neck}/\lambda_{x})B_{x}}\\ \beta_{x}\;=\;\frac{\lambda_{x}}{D_{x}}\frac{\cosh(l/\lambda_{x})}{\sinh^{2}(L_{head}/\lambda_{x})B_{x}}\left[\cosh(l/\lambda_{x})-\frac{1}{2}\sinh(2L_{head}/\lambda_{x})\right]\\ B_{x}\;=\;\coth(L_{head}/\lambda_{x})+{\left(r_{head}/r_{neck}\right)}^{2}\tanh(L_{neck}/\lambda_{x})\\ A_{x}\;=\;\frac{\alpha_{x}{\left(r_{\text{neck}}/r_{\text{dend}}\right)}^{2}P_{x}}{1+(\lambda /2D){\left(r_{\text{neck}}/r_{\text{dend}}\right)}^{2}Q_{x}}-\frac{2D}{\lambda }\beta_{x}\\ B_{x}\;=\;\frac{2\alpha_{x}{\left(r_{\text{neck}}/r_{\text{dend}}\right)}^{2}P_{x}}{1+(\lambda /2D){\left(r_{\text{neck}}/r_{\text{dend}}\right)}^{2}Q_{x}} \end{array}$$

Source code for the analytic solutions and for the simulations is available in ModelDB (McDougal et al., 2017) at modeldb.yale.edu/267050.

## Results

In order to maintain memory and the ability to persistently perform learned tasks over long periods of time, synaptic plasticity must generate stable changes in synaptic efficacies. How this is done is not obvious since the changes in protein number and function are typically transient because of protein turnover and diffusion. One way to obtain such long time scales is through bistable or multistable molecular switches (Lisman, 1985; Lisman and Zhabotinsky, 2001; Aslam et al., 2009; Jalil et al., 2015). We have argued that such a switch is most likely implemented at the level of translation, and that the synthesized protein that potentiates synaptic transmission is PKM*ζ*. The approach we take is based on the observation that its synthesis is likely local in dendrites and perhaps spines (Muslimov et al., 2004; Westmark et al., 2010; Palida et al., 2015), and that the induction of L-LTP results in a local and sustained increase of PKM*ζ* in spines (Hsieh et al., 2021). The synthesis of a specific protein (for example, PKM*ζ*) could affect its own level of translation, and this positive feedback loop can generate bistable or multstable switches (Westmark et al., 2010). Much of our results here though are general and could apply to other forms of a molecular switch, for example, those dependent on posttranslational modifications (Lisman, 1985; Lisman and Zhabotinsky, 2001).

A molecular switch for maintaining synaptic efficacies, must operate in a synapse-specific manner to maintain the computational power of the neural circuits. Until now most models of such a molecular switch were single compartment models that did not analyze the effect of diffusion on synapse specificity. Diffusion, however, could impair synapse specificity because proteins synthesized by one switch could diffuse to a neighboring one and trigger protein synthesis (or phosphorylation) at that location too. It is also thought that maintenance-related proteins have long lifetimes (Heo et al., 2018). Such long lifetimes, as shown below, however, are potentially detrimental to synapse specificity.

Using the model outlined above of bistable switches in dendritic shafts or spines we have calculated the conditions necessary for synapse specificity during the maintenance phase of synaptic plasticity. In general, we use the most rigorous test for synapse specificity by requiring that all synapses can remain independent under all conditions. Mathematically this means that one should be able to maintain a single synapse in the down-state when all others are in the up-state. Other, more lenient, definitions of synapse specificity imply that under some conditions there will be no synapse specificity. We explore the consequences of less rigorous definitions as well.

### Switches in dendrites

To determine when a synaptic site remains isolated from neighboring potentiated synapses, we consider the situation of an inactive switch flanked by 2*N* active switches separated from each other by a distance *L* (see Discussion). This situation is illustrated in Figure 4*A* for the case of *N *=* *2.

As described above, synthesized proteins can diffuse to neighboring sites leading to an increase in protein concentration at the location of the inactive switches, which could result in its activation. Figure 4*A*, lower panel, shows the calculated steady-state spatial profile of protein concentration along the length of the dendritic shaft, computed using NEURON. As expected, the protein concentration peaks at the location of the active switches. In the case illustrated here the distance separating the switches is such that the protein concentration at the site of the inactive switch (x = 0) is low enough that it does not lead to its activation. However, the situation changes when the distance between the switches decreases, as illustrated in Figure 4*B*. When the distance between switches is short enough, the inactive switch that has not been externally activated becomes activated. In this example an activation pattern (e.g., memory) that involved a central inactivated synapse and only four activated neighboring synapses are shown, but the inactivated synapse becomes active by the effects of diffusion, losing its synapse specificity.

As described in Discussion, as the distance *L* decreases, the number of fixed points of Equation 6 changes from two to one. Figure 4*C* shows a plot of the value of the stable (black solid lines) and unstable (black dashed lines) fixed points as a function of the distance *L*. The vertical red dashed line indicates the distance at which a transition occurs from a bistable to a monostable region. This line defines the quantity *L_crit_*.

Figure 4*C* shows that for distances larger than *L_crit_*, there are two stable fixed points. The upper branch of the bistable region corresponds to the situation when all switches are active. In contrast the lower branch represents the situation described in which a synapse can stay weak although all its neighbors are potentiated. When the distance is smaller than *L_crit_* the only stable solution is for all switches to be active, thus there is a loss of synaptic specificity.

The value of *L_crit_* depends on several parameters, as can be inferred from Equation 6. However, we will focus here on the effect of the characteristic length constant, *λ*, since this incorporates the effects of both diffusion and degradation (defined in Discussion as $\lambda =\sqrt{D/K}$). Figure 4*D* shows this dependence. The red dashed-line separates the monostable and bistable regions and corresponds to the value of *L_crit_* for different values of *λ*.

In general, we see that with switches in the dendrite the characteristic value of *L_crit_* (dashed red line) is on the order of 10^2^ μm for proteins that degrade slowly. For example, using a diffusion coefficient *D *=* *10^–3^ μm^2^/ms, typical for proteins the size of PKM*ζ*, and proteins degrading with a time constant of ∼5 h, the corresponding value of *λ* is 120 μm. In this case, the critical distance is on the order of 150 μm, which is a hundred times larger than estimated interspine distances.

An analytical expression for this dependency can be given in the case of Θ being a step-function. By using Equation 6 in the case when $c_{o}<c_{\theta }$, and using the expression for the maximum synthesis rate $I_{o}=fI_{o}^{*}$ (see Discussion), the value of $I_{o}^{*}$ is calculated by Equation 4 to obtain the relationship $L=\lambda \ln(1+2fc_{\theta }/c_{o}^{d})$. Because *L_crit_* occurs when $c_{o}^{d}=c_{\theta }$ and using the value *f *=* *1.25, we obtain $L_{crit}=\lambda \ln(1+2f).$

For the value of *f *=* *1.25 we would get that $L_{crit}=\lambda \ln(3.5)\approx 1.25\lambda$, and since for single spine stability *f *>* *1 we obtain that *L_crit_* > *λ*. Note that when *f* is very close to one, stability in the up-state is very fragile and is likely to be destabilized by small stochastic fluctuations.

### Switches in spine heads

A similar analysis can be made when switches are located in the head of dendritic spines. In this case, the arrangement of the spines is illustrated in Figure 5*A*. Here, an inactive switch is placed in the head of a spine situated at the origin. This spine is flanked by 2*N* other spines with active switches in their corresponding heads.

Proteins synthesized in the spine head diffuse through the spine necks into the dendrite where they can reach the inactive switch. Figure 5*A*,*B*, lower panels, shows the calculated steady-state protein concentration profile in the dendrite. Note that the scale in the axis has decreased by a factor of 100 compared with Figure 4*A*,*B*.

In a similar way to switches in the dendrites take out, that is described in the previous section, as the spines get closer to each other the concentration in the head of the inactive switch increases, and at the critical distance (*L_crit_*) it will cause the switch to become active (Fig. 5*C*). As described in Discussion, the number of stable fixed-points of Equation 8 changes as the distance *L* between spines decreases. The distance at which this transition occurs corresponds to *L_crit_*. In the example shown in Figure 5*C*, the theoretical results are compared with simulation results (circles) using the 1D reaction diffusion package on Neuron (McDougal et al., 2017). In this example the transition occurs at *L_crit_* = 12.85 μm. This value is an order of magnitude smaller than values obtained when the switches are in the dendrites. However, this value depends on the system parameters, as explored in detail below.

An analytical expression for *L_crit_* can be derived under the assumption of a step-function activation:

$$L_{crit}=\lambda \ln\left(1+f\;\frac{B}{A}\right).$$

The functions A=A(λ,D) and B=B(λ,D) are provided in Discussion, and both depend on *λ* and on *D*. Therefore, this relationship is nonlinear in *λ* and depends explicitly on *D* as well.

In Figure 5*D*, we show how the value of *L_crit_* depends on *λ*. This plot also shows for comparison the critical distance between spines for various values of *λ* and the corresponding distance for switches located in the dendrite. Note that the axes are logarithmically scaled.

Clearly, for the same characteristic length constant, when switches are located in the head of dendritic spines, the distance between these spines, which provide synaptic specificity, is significantly smaller than that obtained when switches are located in dendritic shafts. As *λ* increases so does the critical distance, and for values of *λ* ∼ 720 μm the distance is orders of magnitude larger than characteristic interspine distances. Typical spine density in neurons has been reported to be approximately ∼1 spines/μm (Kirov et al., 1999; Sala and Segal, 2014). While the experimental values and theoretical limits are quite different, one must note that there are different spine types, and not all of them form functional synapses; this estimate groups different synaptic types together. Moreover, many synapses do not contain switches, so the actual distance between potentially active spines is larger. Yet, this discrepancy might be concerning.

### Confirmation of methodology using 3D simulations

The analytic results and the previous simulations were all based on a 1D approximation. This approximation is valid if the longitudinal gradients are far larger than the lateral gradients, i.e., the change is significantly greater along the length of the dendrite than across the diameter of the dendrite. To test this assumption, we ran the simulations in 3D with *N* = 5. As 3D simulations are computationally demanding we only modeled the central spine and its two neighbors in 3D, with four additional 1D spines to provide the boundary conditions. The simulation was conducted with *L *=* *19 μm and *L *=* *20 μm, where *L_crit_* was 19.475 μm from the 1D analytic result.

**Figure 6.**
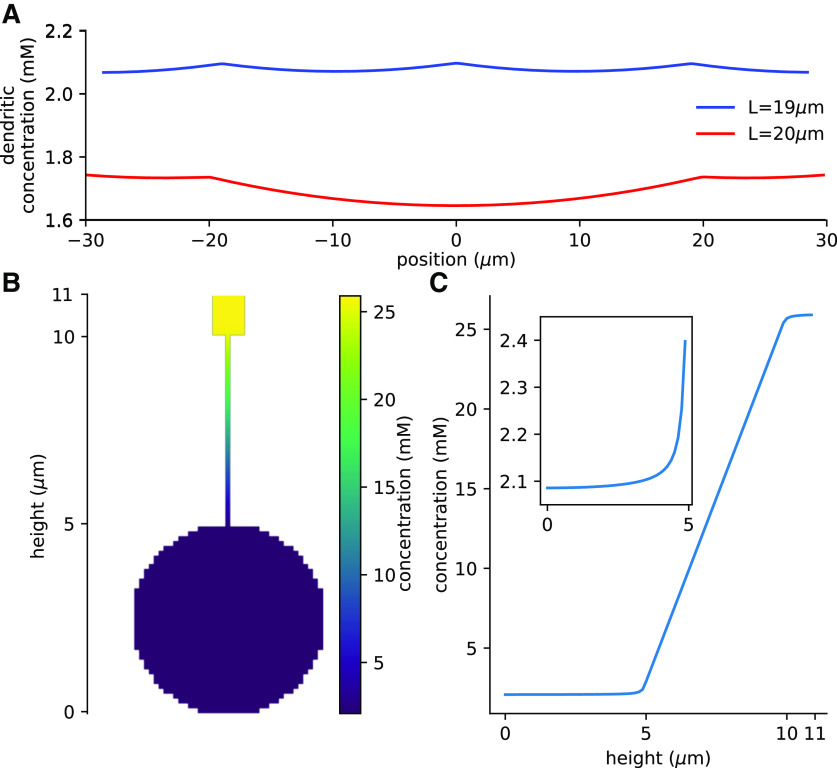
Simulations demonstrate the validity of 1D approximation. ***A***, With neck length 5 μm, *λ* 60 μm, and source 284 μm/s the analytic critical separation distance for the 1D approximation *L_crit_* = 19.475 μm, 3D simulations showed activation of the central spine with a separation distance below this threshold (*L *=* *19 μm) but not above it (*L *=* *20 μm). ***B***, A 2D cross-section of the central activated spine shows large concentration gradient through the spine neck, and uniform activation within the dendrite. ***C***, Plot of the concentrations through the center of the 2D cross-sections shows the concentration is almost homogeneous throughout the dendrite, with a large, linear change along the length of the spine neck. The inset shows a magnified view of the concentration within the dendrite, showing the concentration rapidly stabilizes with distance from the spine.

The results of the 3D simulation confirm the appropriateness of the 1D approximation. In Figure 6*A*, which shows the concentrations averaged over the cross-sections of the dendrite, it is clear the critical distances for activating the central spine are similar in 1D and 3D. This suggests the 1D approximation is valid. To understand why the 1D approximation yields nearly identical results to the 3D simulations, we plotted the concentration across a 2D section that runs through the spine head down to the dendritic compartments (Fig. 6*B*,*C*). The concentration exhibits a rapid linear drop along the length of the spine, but relatively little change in concentration across the dendrite. This is quantitatively displayed shown in Figure 6*C*. Figure 6*C*, inset, shows that the concentration gradient within the dendrite rapidly declines from its value within the spine neck to a nearly constant value. These results show that for these parameters, the detailed 3D simulations produce nearly identical results to the 1D analytical results because of very small gradients across the dendrite.

### Critical distances with a limited number of spines or clusters

Previously we have assumed an infinite number of spines on the dendrite, here we evaluate how limitation on the number of spines can affect synaptic specificity. The infinite limit is the most rigorous definition of synapse specificity, but real dendritic branches have a finite number of spines, so we can relax this assumption. Additionally, we explore whether relaxing the requirement for synaptic independence under all conditions significantly effects the required separation between synapses.

**Figure 7.**
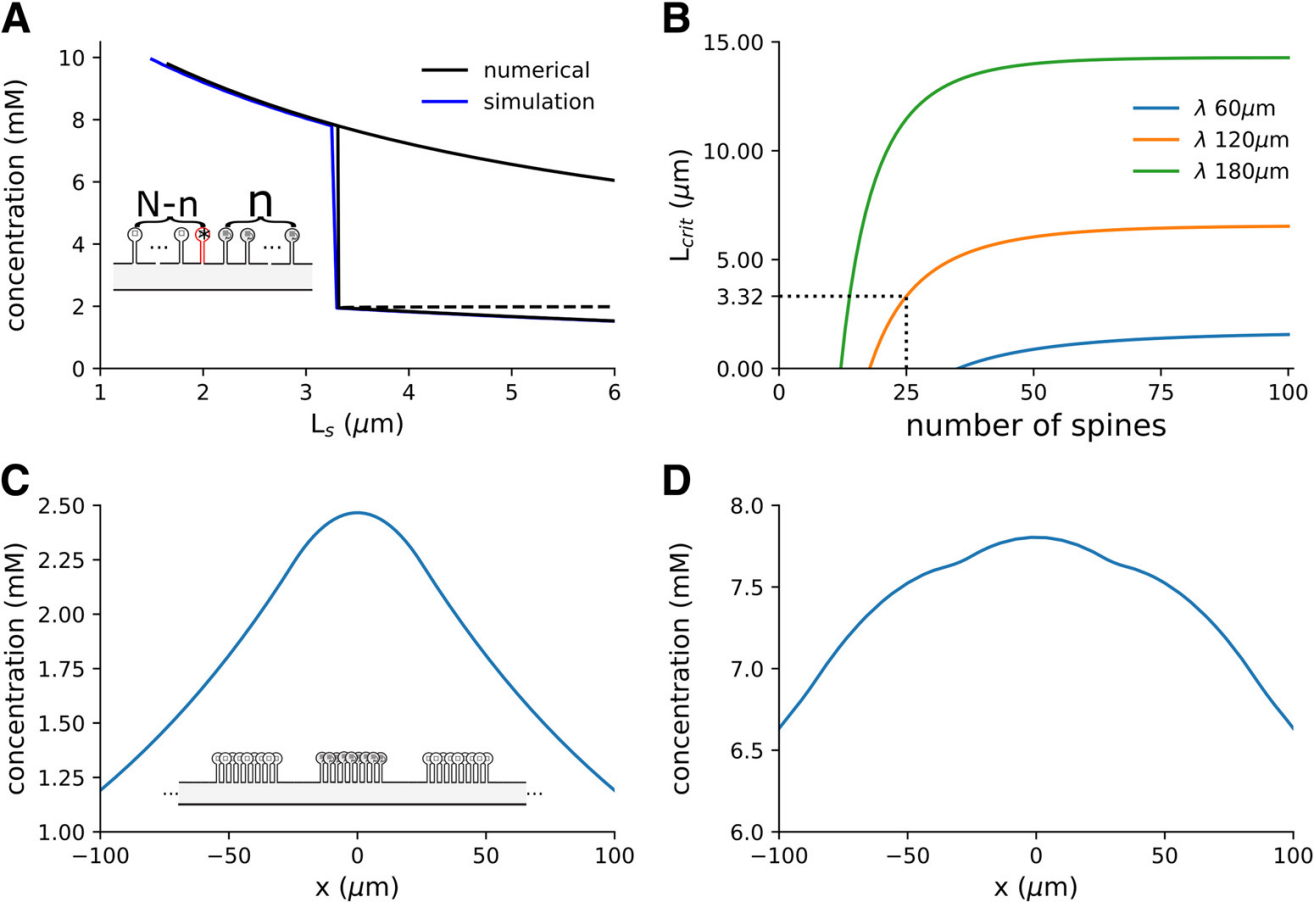
The critical distance depends on the number of potentiated spines, and clustering of spines show similar distance-dependent synaptic specificity. ***A***, Simulation and analytic solutions for a model with *λ* = 120 μm, *N *=* *100 spines of which *n *=* *25 are initially potentiated. The concentrations shown are those in the unpotentiated spine head closest to the potentiated spines (the spine highlighted in red with an * in the schematic). The plot shows a monostable region for *L *<* *3.32 μm where all the spines are potentiated, followed by a bistable region *L *>* *3.32 μm where the spines can remain unpotentiated. The dashed line shows the unstable solution (similar to Fig. 5*C* but with a limited number of spines). ***B***, The critical distance (*L_crit_*) between spines scales with the number of potentiated spines. Here, three examples are shown for different values of the length scale *λ*. The dashed line indicates the model shown in ***A***, with *L_crit_* = 3.32. ***C***, ***D***, Steady-state dendritic concentrations resulting from clusters of 25 spines separated by 2 μm, five clusters are simulated in a dendrite (of which the central 3 are shown) and *λ* = 120 μm (illustrated in the schematic). The central cluster is initially potentiated. ***C***, The distance between clusters is 20 μm, and the neighboring clusters fail to potentiate. ***D***, The distance between clusters is 15 μm, and all the spines become potentiated (note the difference in the concentration scale).

We consider the case where there are a finite number of spines (*N*) on a long dendrite, of which some number *n* are initially potentiated (Fig. 7*A*, inset schematic). With a limited number of spines, the critical distance between spines not only depends on the geometry and diffusion and clearance of the protein, but also on the number of potentiated neighbors. For a given number of initially potentiated spines, there is a minimal interspine distance at which bistability can still be maintained (Fig. 7*A*). In the example in Figure 7*A*, *N* = 100 spines with 25 potentiated and *λ* = 120 μm, bistable solutions occur when the distance between spines exceeds 3.32 μm, and it is possible for spines to remain unpotentiated. For shorter interspine distances (<*L_crit_*) bistability is lost and hence synapse specificity is lost. The minimal distance that supports synapse specificity is a function of the number of initially potentiated synapses as shown in Figure 7*B*. The value of *L_crit_* also strongly depends on the protein’s length scale (Fig. 7*B*, color plots for *λ* = 60, 120, 180 μm).

Another possible form of specificity is between clusters of spines (Scholl et al., 2017), where individual spines within a cluster may not show synaptic specificity, but different clusters could exhibit distinct states of potentiation. We extended the simulation to a model of clusters of spines, where in each cluster there is a limited number of spines and clusters are separated from each other. This model has two characteristic distances; a distance between spines within a cluster and a distance between clusters. For example, we simulated the steady-state dendritic concentration of a model with five clusters of 25 spines, where spines in the central cluster are initially potentiated. There was a distance of 2 μm between spines and a separation between clusters of either 20 μm (Fig. 7*C*), where spines in neighboring clusters remained unpotentiated, or 15 μm (Figure 7*D*), where the spines became potentiated. This shows that as with spines, there is an analogous critical distance for clusters of spines. It also suggests that the intercluster distance required for cluster specificity is inconsistent with observed distributions of spines. As before, these distances depend both on the geometry of the model (Table 1), as well as the diffusion and clearance of the protein.

### Dependence of critical interspine distance on spine morphology

The morphology of the spine head and neck can play a role in *L_crit_*. There are numerous reports showing a persistent change in the volume of spine heads in potentiated spines (Lee et al., 2009; Govindarajan et al., 2011; Tønnesen et al., 2014). We explored here the effects on *L_crit_* for various head sizes. Our results show that changes in the spine head’s volume have negligible effect in the critical distance (data not shown).

**Figure 8.**
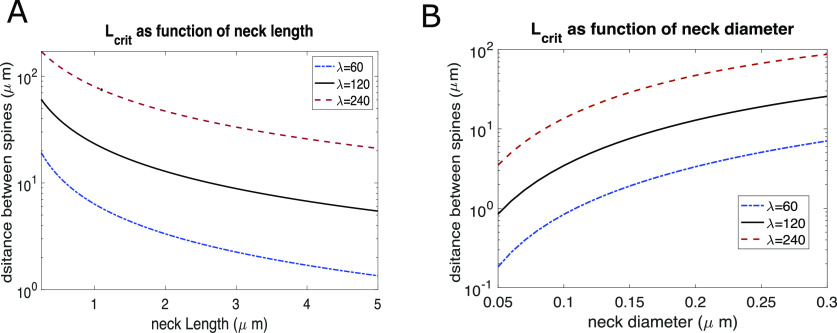
Dependence of *L_crit_* on spine morphology. ***A***, *L_crit_* as a function of spine neck length. ***B***, *L_crit_* as a function of spine neck diameter.

In addition to the above morphologic changes, there is evidence of changes in spine’s neck length (Araya et al., 2014). Although there are reports of a shortening of the spine neck in potentiated spines during LTP induction (Tønnesen et al., 2014), we explored both the effect of increasing and decreasing the length of the spine neck. Our results show that spine neck length plays a significant role in establishing the value of *L_crit_*. Figure 8*A* shows that as the length of the spine neck increases the value of *L_crit_* decreases slowly. Interestingly, a reduction of the spine neck length suggests that the inactive spine can remain isolated only at significantly larger distances. Although the distances explored here seem beyond the typical mean range of spine neck lengths, recent results indicate that there is a large diversity of spine length, that the distribution is non-Gaussian with a large Kurtosis and that spine neck lengths depend on the spine type (Ruszczycki et al., 2012; Kashiwagi et al., 2019).

Additionally, the diameter of the spine neck seems to have a major effect in *L_crit_*. The diameter of the spine neck has been found to be regulated by neuronal and synaptic activity (Bloodgood and Sabatini, 2005; Tønnesen et al., 2014). Various experiments show a slowing of molecules passing through the spine neck as a result of high neuronal activity (Nishiyama and Yasuda, 2015); however, there is evidence of the opposite effect as well (Tanaka et al., 2008; Tønnesen et al., 2014; Nishiyama and Yasuda, 2015). Our results are shown in Figure 8*B* and illustrate that a reduction of the spine neck diameter leads to a significant decrease in the critical distance between spines. Although this parameter has the largest impact of those we have observed, it is technically quite hard to estimate experimentally.

### The implications of different diffusion coefficients in active and inactive spines

The critical distance between spines, *L_crit_*, can be affected by various properties of the dendritic spines as can be inferred from Equation 8 in Discussion. Here, we explored the effects of having a slower diffusion coefficient in the spine than the in the dendrite. Note that changes in these parameters will affect the ability of an isolated active spine to remain in the up-state; therefore, we recalculated the magnitude of the maximum synthesis rate $I_{o}^{*}$ for each data point (see Eq. 5).

The presence of actin in the cytoskeleton (Matus, 2000; Shirao and González-Billault, 2013) of the spine can slow the diffusion of proteins compared with conditions in the dendrite, effectively decreasing the diffusion coefficient inside the spine. It is also possible to hypothesize that the diffusion inside a potentiated spine might be different from in an unpotentiated spine (Nishiyama and Yasuda, 2015). Such a change in the diffusion might be a consequence of structural changes in spines which occur after the induction of LTP (Fukazawa et al., 2003). We have derived an equation that can account for how *L_crit_* depends on these local changes in diffusion coefficients, which has the following form:

$$L_{crit}=\lambda \ln\left(1+f\frac{B_{a}}{A_{x}}\right).$$

The function $B_{a}$ is defined in and assumes constants consistent with an active spine. The function $A_{x}$, also defined in, is either for the active (*x *=* a*) or inactive (*x *=* i*) spines. The inactive spine case ( $A_{i}$) assumes that an isolated inactive spine can be stable in the UP state without further structural changes, whereas the active spine case ( $A_{a}$) is based on the assumption that an isolated spine will only be stable once further structural changes are induced.

**Figure 9.**
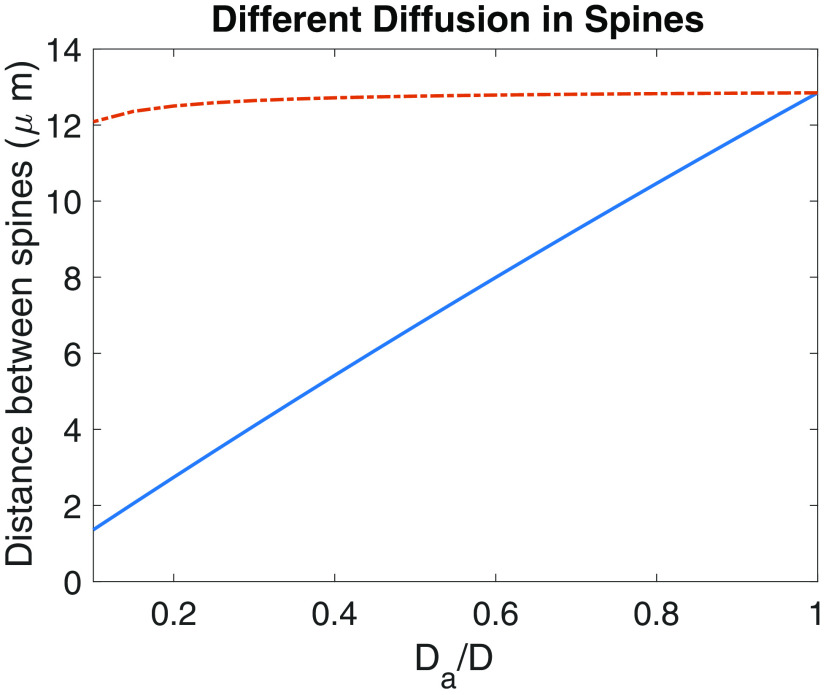
Dependence of *L_crit_* on the ratio between the diffusion coefficient in the dendrite *D* and on the diffusion coefficient in the active spine *D_a_*. The dash-dot red line is based on the assumption that the critical current is consistent with an inactive spine, and the solid blue curve assumes a critical distance consistent with an active spine.

The results for these two different assumptions are shown in Figure 9 and have been confirmed by simulations (data not shown). These results show that if we assume a critical current ( $I_{0}^{*}$) consistent with an inactive spine, the different diffusion coefficients have minimal effect on the critical distance (Fig. 9, dash-dot red line). If on the other hand we assume a critical current consistent with an active spine, the slower diffusion rates in spines can result in a significantly shorter critical distance (Fig. 9, solid blue line). The difference between these two cases arises because in the latter case a much lower current needs to be assumed to keep active spines in the UP state, and therefore less concentration accumulates in the inactive spine.

### Dependence of critical distance with activation characteristics of the molecular switch

The rate of protein synthesis in our model depends on the local protein concentration. This positive feedback mechanism is described through an activation function Θ(*c* – *c_θ_*). In many results presented here, Θ is assumed to be a step-function. However, in biological systems this function is likely to be less steep. Moreover, in our simulations we have replaced it by a steep Hill-function, with an exponent of *n *=* *40 or *n *=* *300 (see Table 1; Eq. 13).

**Figure 10.**
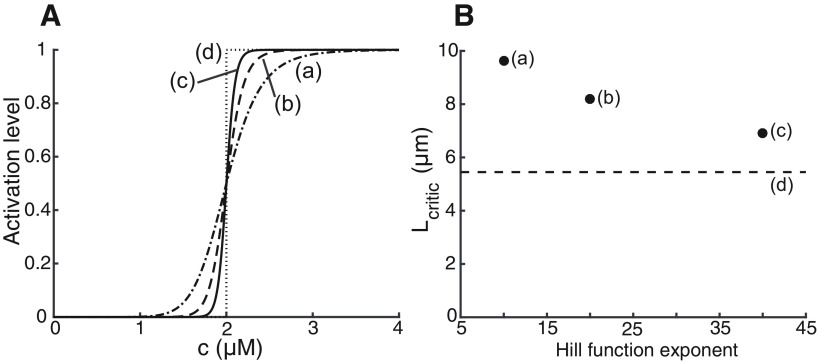
Dependency of critical distance with steepness of activation curve. ***A***, Illustration of the different activation curves used in the simulations presented in panel ***B***. Labels a, b, and c correspond to those of panel ***B***. ***B***, Critical distance between spines increase as the steepness of the activation curve Θ decreases. Results were obtained for the standard parameters (see Table 1) and characteristic length constant *λ* = 120 μm. Points labeled a, b, and c correspond to values of the exponent *n* in Equation 13, equal to 10, 20, and 40, respectively. Point d represents the result using a step-function as activation curve.

Here, we explore the effect of relaxing the sharp steepness condition and determine the effect of decreasing the activation slope (i.e., decreasing *n*) in the critical distance *L_crit_*. We apply this to the case when switches are located in spine heads and assume that all active spines are still operating near the saturation regime. Consequently Eq. 8 is still valid except that the function Θ has been modified. Figure 10*A* illustrates the changes in the shape of the function Θ corresponding to different values of *n* (as defined in Eq. 13). The effect of these different values of *n* on *L_crit_* are shown in Figure 10*B*. The dashed line is the case of using a step-function as the activation curve. The results show a dependence with the slope of the activation curve. A decrease in the slope by a factor of four increases the critical distance by ∼2.5 μm. These changes are relatively small, showing that our results do not depend strongly on the steepness of the activation function (here we have used longer neck length of 5 μm because it accentuates the relative effect of the step function).

## Discussion

The maintenance of long-term changes in synaptic efficacies of activated synapses most likely requires the ongoing synthesis or activation of specific synaptic proteins. Such proteins remain active for long periods of time and might be degraded slowly. These proteins will diffuse and potentially reach the site of other synapses not previously activated and potentially activate them, thus compromising the specificity of the initial activation pattern. Here, we explored, using computational and analytical methods, the conditions under which synapses can remain isolated from each other during the maintenance phase of synaptic potentiation or L-LTP.

In order to provide a measure of the isolation of a synapse, we calculated the minimum distance (*L_crit_*) between synapses that would leave a synapse in an unpotentiated state while all other synapses around it are potentiated. In order to do this, we used a simple bistable switch to model the process comprised of positive-feedback onto protein synthesis and a degradation mechanism. In our setting the location of a switch corresponded with the location of a synapse, whether activated or not, and these switches were immobile. The main quantity representing the combined effect of diffusion and degradation of proteins is the characteristic length constant *λ* (see Eq. 3 in Discussion), which can be thought of as the distance over which the concentration decays to ∼36% (i.e., 1/*e*) of its magnitude at the (point)source (see Eq. 4 in Discussion).

Our main result is presented in Figure 5*D*. Here, we show a comparison of *L_crit_* as a function of the characteristic length constant *λ* for two scenarios of the location of the switch: in dendritic shafts or in dendritic spines. Our calculations clearly show that the synthesis of proteins inside dendritic spines is necessary for synapse isolation. Here, distances of <1 μm can be achieved for values of *λ* < 60 μm, equivalently to degradation times of the order an hour. In contrast for these same values of *λ*, the minimum distance obtained when protein synthesis occurs in dendritic shafts is ∼100 μm. Note that the observed distance between spines is on the order of a few micrometers, and synapse specificity during the induction of L-LTP has been experimentally observed at the scale of a few micrometers (Govindarajan et al., 2011). Our analysis also makes it clear that potentiating a single synapse and observing if neighbors are potentiated (Govindarajan et al., 2011) is a weak test of synapse specificity, and that a better test is to potentiate several synapses that surround an unpotentiated synapse.

We conclude from this that in order for synapses to remain isolated from each other during the maintenance phase of memory, it is necessary for the molecular switch to be inside dendritic spines (cf. Bourne et al., 2007). These results also imply that in synapses that lack spines such as inhibitory synapses, this method of long-term maintenance cannot provide the same measure of synapse specificity. On the basis of our results we predict that if activated synapses in dendritic spines express L-LTP, for instance by showing an increase in the level of PKM*ζ* (Palida et al., 2015) or other relevant proteins, then these spines should also contain polyribosomes (for a protein synthesis-dependent switch).

Slowly degrading proteins like PKM*ζ* or PKC*ι* /*λ*, which have degradation times of ∼4 h (Palida et al., 2015) or longer have values of *λ* ∼ 120 μm (assuming a diffusion constant as in Table 1). On the other hand to obtain synapse specificity at observed characteristic interspine distances of ∼1 μm, λ would need to be on the order of 20 μm, which is not consistent with the observed time scale of PKM*ζ* turnover (Vogt-Eisele et al., 2014).

This presents a challenge for the theory, since for these proteins the critical distances would be relatively large (Fig. 5*D*) and thus would not offer good isolation. However, if the maintenance-related proteins have active and inactive forms, and the active form can degrade into an inactive form (cf. Harvey et al., 2008; Lee et al., 2009), then this could lead to a shorter effective *λ* for the active form. Because the activity of PKM*ζ* depends on its phosphorylation state (Jalil et al., 2015) it is possible that if PKM*ζ* is dephosphorylated this might cause a reduction in its active lifetime (i.e., larger *K*) and therefore reduce the value of *λ*. Thus, our results would indicate that for slowly degrading proteins involved in L-LTP, like PKM*ζ*, to also satisfy conditions for synapse isolation, their active form should be found primarily in activated dendritic spines and not in dendritic shafts. Our previous model of PKM*ζ*-dependent maintenance was more complex than the simple switch used here (Jalil et al., 2015), and on the basis of experimental observations, included two phosphorylation sites on the PKM*ζ* protein that control its activity. In such a model the relevant length scale is of the active-phosphorylated form of the protein, which is likely to have a shorter length-scale than total protein concentration. However, if experiments reveal that the key “memory molecule” (PKM*ζ*, phosphorylated PKM*ζ*, or in general any other molecular state identified) does not have a sufficiently short length constant, this will pose a fundamental challenge to a maintenance theory based on positive feedback.

Here, we also explored how diffusional and morphologic characteristics of the dendritic spine might improve conditions for synaptic isolation. Results are shown in Figure 8*C*,*D* and illustrate that there is a significant decrease in *L_crit_* as the diameter of the spine neck becomes smaller or its length becomes larger. This suggests that a further condition necessary for synaptic isolation would be that synapses at dendritic spines undergoing L-LTP would have narrower spine necks. Conditions on the spine neck width are supported by experimental evidence showing that the key regulatory component of the dendritic spine is the cross-section of the spine neck and examples of spines with high restrictive necks have been observed (Bloodgood and Sabatini, 2005; Tønnesen et al., 2014). Although changes in spine neck diameter have been reported to occur also in the opposite direction during induction (Tønnesen et al., 2014), it has not been verified experimentally if this trend remains or reverses during L-LTP. However, these condition which further isolate synaptic spines also pose a problem as this might also lead to making the synapse electrotonically isolated, and hence actually reduce the magnitude of the EPSP in the cell body.

Structural changes to synapses as a result of L-LTP might not only affect their morphology, but also their diffusional properties through changes in actin structures (Fukazawa et al., 2003; Borovac et al., 2018). We explored how this might impact synapse specificity (Fig. 9). We have shown that such changes can actually enhance synapse specificity (Fig. 9, blue curve), but only for a specific assumption. For synapses after L-LTP in which the diffusion is slower, the actual critical current required to obtain bistability in an isolated spine is lower. If we assume this lower critical current, then the critical distance is smaller as well (Fig. 9, blue curve); on the other hand, if we assume a critical current consistent with fast diffusion that presumably exists before L-LTP, then the critical distance is similar (Fig. 9, dash-dot red line). What this implies is that the early phase of L-LTP must be long enough such that structural changes occur before it ends. This is quite reasonable because the early phase of L-LTP can last for several hours, and structural changes are likely to occur on a faster time course (Fukazawa et al., 2003). Indeed, recent results indicate that changes in F-actin occur within minutes after the induction of LTP (Goto et al., 2021). This scenario also might explain why such structural changes might be necessary for the maintenance of L-LTP.

Synaptic specificity has been verified experimentally during LTP induction (Andersen et al., 1977; Lynch et al., 1977; Harvey et al., 2008), most results are confined to the early phases of LTP. Results presented by Govindarajan et al. (2011) indicate that synapse specificity is preserved in dendrites in which L-LTP is induced. However, our analysis shows that the methodology used experimentally for testing synapse specificity in which a single spine is potentiated and is observed if its neighbors remain unpotentiated, is a weak test of synapse specificity. A better test is potentiating several nearby synapses and testing whether synapses interspersed between them remain unpotentiatied. Indeed, in terms of the model, it is very easy to choose parameters such that a single synapse, even without a spine, can potentiate without affecting its neighbors. In terms of Equations 4, 5, this simply requires choosing a value of *I*_0_ that is slightly above the critical value for a self-maintaining synaptic up-state. In addition, the experimental results of Govindarajan et al. (2011) show that synapses in which L-LTP was induced are capable of facilitating L-LTP in nearby spines stimulated 40 min later even when the second one is stimulated in the presence of protein synthesis inhibitors. This previously observed phenomenon, termed synaptic tagging and capture (Frey and Morris, 1997) suggests that that proteins synthesized in one spine can diffuse into a second one up to distances of ∼70 μm. Although the kinetics and nature of these proteins are not sufficiently characterized, a role for PKM*ζ* in the process of synaptic tagging and capture has been demonstrated (Sajikumar et al., 2005). These results indicate that although long-term plasticity is localized to a synaptic spine, other aspects of protein synthesis-dependent plasticity are less local. In this article, we do not provide a mechanistic basis for aspects of synaptic plasticity that are less local in nature.

Here, it has been assumed that synapse specificity is necessary and desirable. It has been suggested that there are advantages to having less synapse specificity (Govindarajan et al., 2006). Less specificity could generate clusters of strong synapses, which in turn could recruit dendritic spikes and can be used for local dendritic computations. We specifically modeled scenarios with clusters of synapses in which synapse specificity might only be required between clusters but not within clusters (Fig. 7*C*,*D*), and found a minimal critical distance between clusters necessary for maintaining the independence of clusters. We find that already for clusters of moderate size (e.g., 25 synapses in a cluster) large distances between clusters (∼15 μm) are required to maintain cluster independence, and such highly uneven spine distributions with large intercluster separation are not consistent with experimental data.

There is strong experimental evidence to support both the ideas of synaptic specificity and the memory-maintenance role of proteins. In this work we have addressed the question of how the diffusive nature of the latter can impose limits on the former. We propose here that for these two features of synaptic plasticity to co-exist, dendritic spines expressing L-LTP must contain polyribosomes, that the active form of the protein (e.g., the phosphorylated form of PKM*ζ*) has a relatively short length scale, and that synaptic spines have small spine neck diameters. If these conditions do not hold, this would constitute a fundamental challenge to a theory of maintenance based on a bistable, positive-feedback loop.

## Synthesis

Reviewing Editor: Niraj Desai, National Institute of Neurological Disorders and Stroke Decisions are customarily a result of the Reviewing Editor and the peer reviewers coming together and discussing their recommendations until a consensus is reached. When revisions are invited, a fact-based synthesis statement explaining their decision and outlining what is needed to prepare a revision will be listed below. The following reviewer(s) agreed to reveal their identity: NONE. Note: If this manuscript was transferred from JNeurosci and a decision was made to accept the manuscript without peer review, a brief statement to this effect will instead be what is listed below.

I read the manuscript along with the reviews transferred from the Journal of Neuroscience and the comments of that journal’s reviewing editor.

The reviewers offered strong and detailed arguments about the model presented in the manuscript, especially how well it conforms to empirical data. In this latest revision, the authors acknowledge those arguments and respond effectively: this is what the model says and predicts, here are the obstacles it must overcome, and here are ways it might do so.

This isn’t a perfect story -- it’s not wrapped up with a nice bow, and the authors know that -- but it’s a useful contribution to the debate. Even if the mechanism of long-term synaptic modifications turns out to be something else, this is still useful.
